# Optimization of the Coverage and Accuracy of an Indoor Positioning System with a Variable Number of Sensors

**DOI:** 10.3390/s16060934

**Published:** 2016-06-22

**Authors:** Francisco Domingo-Perez, Jose Luis Lazaro-Galilea, Ignacio Bravo, Alfredo Gardel, David Rodriguez

**Affiliations:** Department of Electronics, University of Alcalá, Alcalá de Henares E-28806, Spain; lazaro@depeca.uah.es (J.L.L.-G.); ibravo@depeca.uah.es (I.B.); alfredo@depeca.uah.es (A.G.); david.rodriguez@depeca.uah.es (D.R.)

**Keywords:** sensor placement, indoor positioning, range-difference, evolutionary optimization, multi-objective optimization

## Abstract

This paper focuses on optimal sensor deployment for indoor localization with a multi-objective evolutionary algorithm. Our goal is to obtain an algorithm to deploy sensors taking the number of sensors, accuracy and coverage into account. Contrary to most works in the literature, we consider the presence of obstacles in the region of interest (ROI) that can cause occlusions between the target and some sensors. In addition, we aim to obtain all of the Pareto optimal solutions regarding the number of sensors, coverage and accuracy. To deal with a variable number of sensors, we add speciation and structural mutations to the well-known non-dominated sorting genetic algorithm (NSGA-II). Speciation allows one to keep the evolution of sensor sets under control and to apply genetic operators to them so that they compete with other sets of the same size. We show some case studies of the sensor placement of an infrared range-difference indoor positioning system with a fairly complex model of the error of the measurements. The results obtained by our algorithm are compared to sensor placement patterns obtained with random deployment to highlight the relevance of using such a deployment algorithm.

## 1. Introduction

Sensor placement is an important task in the design of indoor positioning systems, since the amount of sensors and their location affect the accuracy and the cost of the whole system. Among available methods for estimating the position of a target, range-based localization systems use anchor nodes and measurements that can be converted into distances or distance differences; e.g., time of arrival (TOA), time-difference of arrival (TDOA), received signal strength (RSS), *etc*. Once we have obtained the information about the distances between the target and the anchor nodes, we can perform trilateration or multilateration to estimate the actual position of the target. The reader can find a comprehensive review about positioning techniques and technologies in [1]. The methods that we propose in this paper can be applied to any positioning system based on the range-difference of arrival (RDOA). It does not matter if the target is an emitter or a receiver; however, we consider that the target is an emitter and that the anchors are sensors. Henceforth, we refer to anchors and sensors as the same thing.

The major contributions to the accuracy of an RDOA-based system are the quality of the measurements, the target-anchor geometry and the estimation algorithm. We assume that we will use an efficient estimator, *i.e*., the estimator attains the Cramér–Rao lower bound (CRLB), for computing the estimated position; hence, we can optimize the performance limit of the system without restricting ourselves to a single algorithm. We can easily see the effect of the uncertainty of the measurements and the geometry when we look at the intersection of the hyperbolae. A good geometry provides an orthogonal intersection that minimizes the propagation of the measurement uncertainties to the position estimation error [2]. Chen *et al*. [3] found that simple regular shapes minimize the upper bound of the error of the linear least squares algorithm. A recent work [4] highlights the improvement of the accuracy when deploying anchors following a platonic solid scheme—cubes or tetrahedrons—compared to random deployment in a 3D scenario.

The CRLB, which is computed with the inverse of the Fisher information matrix (FIM), is the minimum variance attainable with an unbiased estimator; in this case, the estimator is said to be efficient. Much has been written on the subject of finding geometries that optimize a metric of the CRLB or FIM. Abel [5] uses a geometric interpretation of the CRLB to place sensors along a linear array. This work was able to replicate the results achieved in [6] without extensive algebraic manipulation, computer-aided maximization or the consideration of a particular estimator. Levanon [7] finds the lowest geometric dilution of precision (GDOP) considering absolute ranges or pseudo-ranges. He simplifies the problem assuming constant variances in the error of the measurements. In this case, the GDOP is proportional to the CRLB obtained with Gaussian measurements [8]. The assumption of the constant variances implies that the CRLB depends only on the anchor-target geometry. Then, he determines the lowest GDOP using *N* anchors and shows that it is found in the center of the corresponding regular polygon whose vertices are the anchors. Another work [9] deals with optimum sensor arrays for TDOA localization and derives sufficient conditions to achieve a minimum CRLB. Again, the system is oversimplified with constant and even uncorrelated noise variances. Lui and So [10] reach the same conclusions considering correlated variances; *i.e*., uniform angular arrays provide the best geometry. Martínez and Bullo [11] provide closed-form expressions for the determinant of the FIM in 2D and 3D scenarios and analyze the set of points that yield global optima in the 2D case. The same metric is considered in [12] to minimize the volume of the uncertainty ellipsoid for range, TOA and bearing localization under the assumption of a constant noise variance, which is equal for all of the sensors. A similar approach is discussed in [13] for TDOA considering both equal and unequal variances, though constant. They conclude that considering unequal variances requires numerical methods for solving the problem. Meng *et al*. [14] study the same problem when using combinations of different types of sensors. Most of these works consider sensor placement to improve the localization of a known source. On the other hand, Isaacs *et al*. [15] consider an uncertain target location in a TDOA problem. They conclude that the sensor-target range affects the localization performance, and they find that arrays of sensors with the same angular separation provide the best geometries. Ho and Vicente [16] study the problem when the target is distant and find good geometries with the shape of rings and sensors in the center.

The works described above focus on the localization of a single source, whereas in a more realistic situation, we are interested in placing the sensors to cover an area. Moreno-Salinas *et al*. [17] extend the work of [11,12,15] to multiple targets. They use numerical methods to maximize the sum of the logarithms of the FIMs of each target and obtain values very close to the theoretical optimum. Neering *et al*. [18] compute the CRLB of several grid points of the region of interest (ROI) as candidates for the target position and minimize a weighted sum using a gradient descent method. The weights allow one to give priorities to some positions of the area. Jourdan and Roy [19] present an iterative method that places sensors to minimize the position error bound, which is a metric introduced in [20] computing the square root of the trace of the CRLB. They consider very realistic scenarios, but the amount of sensors is fixed and their positions constrained to lie on boundaries. In addition, they only optimize one criterion. Perez-Ramirez *et al*. [21] find the optimization of an averaged metric over a grid to have the problem of hiding spots with poor localization performance. Their proposal involves the minimization of the minimum Fisher information matrix, *i.e*., the worst case, with an iterative method. The error model under consideration is distance dependent, and they deal with a 3D scenario. Additionally, they consider the use of heterogeneous sensors and the placement of sensors in an area where there are other fixed sensors. However, they minimize a single objective, such as the volume of the error ellipsoid, which does not provide information about the shape of the error ellipsoids. Their approach deals only with a fixed number of sensors.

Recent approaches solve the sensor placement problem with sensor selection and convex optimization. Joshi and Boyd [22] propose a heuristic for selecting a subset of sensors out of a higher number of candidates for linear measurement models. Chepuri and Leus [23] deal with the same problem considering non-linear measurement models with independent observations. They use the additive property of FIM for independent measurements and place sensors so that a predefined accuracy is achieved with a given probability. On the contrary, we do not need to choose a desired accuracy, nor do we need to constrain the position of the sensors to a grid. Another approach involves leveraging the submodular property of some performance metrics [24]. The advantage of optimizing a submodular function is the availability of simple algorithms that are near-optimal. Shamaiah *et al*. [25] use a greedy algorithm for solving the sensor placement problem in a linear dynamical system. Their work shows that the greedy algorithm outperforms convex relaxation in both accuracy and computation time. As for non-linear models, Rao *et al*. [26] linearize the range-only problem and use a greedy algorithm to optimize two submodular functions.

After reviewing the state-of-the-art of sensor placement for localization, we have reached the conclusion that we are bound to resort to heuristic methods when deploying sensors in a complex scenario, considering complex functions for the measurement error model or focusing on a whole ROI instead of a single target. In this paper, we apply multi-objective evolutionary optimization to obtain the optimum sensor placement. Inspired by the work of Chaudhry *et al.* [27], we have adapted the well-known non-dominated sorting genetic algorithm (NSGA-II) [28] to solve the sensor placement problem for target localization. This paper continues our previous works [29,30], where we used a standard multi-objective genetic algorithm to place sensors considering multiple criteria. In [29], we placed a fixed number of sensors for localization with range-difference measurements while considering several criteria related to accuracy, whereas in [30], we considered a variable amount of sensors, as well as non-line of sight (NLOS) conditions. This contribution introduces the problems that appear when applying the algorithm without modifications considering a variable number of sensors. We opt to modify the original NSGA-II adding speciation and evolving subpopulations according to the size of different sensor sets. Results show a considerable improvement over standard NSGA-II. Overall, we can summarize the global advantages of our work compared to those works in optimum sensor placement for target localization:

- The multi-objective optimization of different metrics from the CRLB: Most of the related work deals with the determinant of the FIM. This metric is related to the volume of the error ellipsoid. However, an elongated ellipsoid may result in a small volume, whereas the error in the major axis is high.
- We do not constrain the position of the sensors.
- The consideration of obstacles that can cause occlusions to NLOS sensors: we must therefore maximize the coverage of the ROI.
- The number of sensors can vary within an interval. Searching solutions with high accuracy, but a low amount of sensors is also an objective.
- Since we optimize conflicting objectives, we obtain a set of Pareto optimal solutions. We find this to be the greatest advantage of multi-objective optimization, since we obtain every optimal solution and know the values of the objectives. This information can be used by the resource manager according to the current needs and availability. To the best of our knowledge, there are not any other researchers that address the sensor placement problem for localization this way. A comprehensive review of multi-objective optimization applied to sensor networks was recently published [31]. Most of the works referenced in the survey focus on sensor deployment for optimizing coverage and energy management, and those that deal with target tracking just address the sensor scheduling problem [32,33].

The rest of the paper is organized as follows. Section 2 gives a quick overview of both the positioning problem with range-difference measurements, in Section 2.1, and the objectives under consideration for sensor placement, in Section 2.2. We describe our algorithm in Section 3 and present some results in Section 4. Finally, Section 5 provides the conclusion and some remarks for future work.

## 2. Problem Statement

This section starts with a short overview of RDOA position estimation, which ends focusing on the uncertainty of the localization error. The reader is referred to [34] for the basic mathematics of position estimation. After introducing the localization problem, we describe our approach for sensor placement. The objective functions under consideration are presented at the end of the section.

### 2.1. Position Estimation

Let us assume that we have *K* range measurements from which we can pick K-1 sensors and subtract their measurement values from the value of the remaining sensor, which acts as a reference. It is irrelevant which sensor is picked as the reference since we take into account the mathematical correlation of the measurements appropriately and can carry out outlier detection at the level of the undifferenced observations [35]. The K-1 range-difference measurements ${\hat{d}}_{i,r}$ can be expressed as a function of the position of the target $x_{T}$:

(1) $$\begin{array}{ccc} {\hat{d}}_{1,r} & = & ∥x_{T}-x_{1}∥-∥x_{T}-x_{r}∥+\epsilon_{1,r};\\ {\hat{d}}_{2,r} & = & ∥x_{T}-x_{2}∥-∥x_{T}-x_{r}∥+\epsilon_{2,r};\\ & \vdots & \\ {\hat{d}}_{K-1,r} & = & ∥x_{T}-x_{K-1}∥-∥x_{T}-x_{r}∥+\epsilon_{K-1,r} \end{array}$$

Variable $x_{i}$ is the coordinate vector of sensor *i*, where *i* takes values from one to K-1. The subscript *r* denotes the reference sensor. Operator $\left|\left|\cdot \right|\right|$ represents the $\ell^{2}$ norm, and $\epsilon_{i,r}$ is the distance difference measurement error of the *i* and reference sensors. Assuming normal distribution, the range-difference measurement is modeled as ${\hat{d}}_{i,r}∼N\left(d_{i,r},\sigma_{i}^{2}+\sigma_{r}^{2}\right)$. The mean $d_{i,r}$ is the true range-difference, and the variance is the sum of the variances of the distance measurement between sensor *i* and the target, denoted as $\sigma_{i}^{2}$, and the reference sensor and the target, denoted as $\sigma_{r}^{2}$. Problem (1) can be solved using iterative or closed-form methods; the most popular ones are reviewed in [36].

As far as the uncertainty of the estimation is concerned, the minimum variance that an unbiased estimator can attain is given by the CRLB, calculated with the inverse of the FIM [37], which is denoted as I. The expression of the CRLB for the RDOA problem is [38]:

(2) $$I^{-1}={\left(G^{⊤}\Sigma^{-1}G\right)}^{-1}$$

where the superscript ⊤ represents the transpose operator. The matrix G is formed by the differences of the partial derivatives of the norms from Equation (1), *i.e*., differences of unit vectors. In the 2D case, G takes the form:

(3) $$G=\left[\begin{array}{cc} \frac{x_{T}-x_{1}}{∥x_{T}-x_{1}∥}-\frac{x_{T}-x_{r}}{∥x_{T}-x_{r}∥} & \frac{y_{T}-y_{1}}{∥x_{T}-x_{1}∥}-\frac{y_{T}-y_{r}}{∥x_{T}-x_{r}∥}\\ \frac{x_{T}-x_{2}}{∥x_{T}-x_{2}∥}-\frac{x_{T}-x_{r}}{∥x_{T}-x_{r}∥} & \frac{y_{T}-y_{2}}{∥x_{T}-x_{2}∥}-\frac{y_{T}-y_{r}}{∥x_{T}-x_{r}∥}\\ \vdots & \vdots \\ \frac{x_{T}-x_{K-1}}{∥x_{T}-x_{k-1}∥}-\frac{x_{T}-x_{r}}{∥x_{T}-x_{r}∥} & \frac{y_{T}-y_{K-1}}{∥x_{T}-x_{K-1}∥}-\frac{y_{T}-y_{r}}{∥x_{T}-x_{r}∥} \end{array}\right]$$

Finally, having a common reference for all of the RDOA measurements implies that the reference error is present in every RDOA measurement; thus, the covariance matrix Σ is fully populated:

(4) $$\Sigma =\left[\begin{array}{cccc} \sigma_{1}^{2}+\sigma_{r}^{2} & \sigma_{r}^{2} & \cdots & \sigma_{r}^{2}\\ \sigma_{r}^{2} & \sigma_{2}^{2}+\sigma_{r}^{2} & \ddots & \vdots \\ \vdots & \ddots & \ddots & \vdots \\ \sigma_{r}^{2} & \cdots & \cdots & \sigma_{K-1}^{2}+\sigma_{r}^{2} \end{array}\right]$$

The following subsection presents the objectives considered for optimum sensor placement.

### 2.2. Sensor Placement

In order to obtain an optimum sensor deployment scheme, we focus on three design criteria, namely the number of sensors, accuracy and coverage. We aim to develop an algorithm that automatically finds optimal deployment patterns that minimize the number of sensors in use and the uncertainty of the position estimation while maximizing the coverage. There is obviously a trade-off among these criteria, since the accuracy and coverage improve as long as the number of sensors increases. Different accuracy measures are also in conflict with each other. Thus, we do not obtain a single optimum solution, but a set of optimal solutions, the so-called Pareto front (PF). Selecting one of these Pareto-optimal solutions involves the application of high-level criteria. The advantage of finding the Pareto front of the sensor deployment problem is the fact that the resource manager knows every possible solution; therefore, he can select one of them according to the current needs. To summarize, we will use the algorithm presented in Section 3 to solve a multi-objective optimization problem. The objectives under consideration are described in this section. The decision variables are the coordinates of the sensors. The amount of decision variables changes according to the number of sensors. Since the sensors cannot be deployed out of the ROI or within obstacles, our constraints are these boundaries. The last constraint is the amount of sensors, which is given by two values representing the minimum and the maximum number. We detail the actual parameters of the problem at the beginning of each example of Section 4.

#### 2.2.1. Accuracy Objectives

We do not focus on a particular estimator for solving Problem (1), but we assume that an efficient estimator that attains CRLB will be used. A sensor set that obtains a low CRLB usually obtains a low MSE of the estimate of the target position as well [23]. Several scalar metrics of the CRLB can be obtained [39], and each one of them has a different geometric interpretation related to the error ellipsoid. In previous work [29], we have placed a fixed number of sensors considering some of these performance measures at the same time instead of focusing on a single metric. This approach allows one to have a better knowledge of the estimated position. If we only minimize the trace of the CRLB, which means minimizing the MSE, we cannot know anything about the shape of the error ellipsoid. However, we can combine the trace with another metric related to the circularity of the ellipse. Therefore, we avoid obtaining an estimate of the position with an elongated ellipse while keeping the MSE to a minimum. This can be achieved with a multi-objective optimization of the following functions:

(5) $$f_{\text{MSE}}=\sum \mathrm{eig}\left(I^{-1}\right)$$

(6) $$f_{\text{circ}}=\frac{\max\left(\mathrm{eig}(I^{-1})\right)}{\min\left(\mathrm{eig}(I^{-1})\right)}$$

The eigenvalues of a covariance matrix are proportional to the length of the axes of the ellipsoid. Equation (5) is equivalent to the trace of $I^{-1}$, which is the MSE. Dividing the maximum eigenvalue by the minimum provides a measure of the circularity; see Equation (6). The goal is to keep $f_{\text{circ}}$ as close to one as possible, without incrementing the value given by $f_{\text{MSE}}$.

After defining the ROI where the localization of the target takes place, we select *P* test points as candidates for the true localization of the target. A regular square or cubic grid is considered for obtaining these points. After obtaining the *P* evaluations of the performance measures, we need a scalar value related to the evaluation of the metric for the whole region. According to the requirements of the resource manager, it could be interesting to focus on the worst case or on the average uncertainty of the region:

(7) $$f_{\text{worst}}=\max\left(\overset{P}{\underset{i=1}{⋃}}\left(w_{i}f_{i}\right)\right)$$

(8) $$f_{\text{avg}}=\frac{\sum_{i=1}^{P}\left(w_{i}f_{i}\right)}{\sum_{i=1}^{P}w_{i}}$$

The function $f_{i}$ could be any of Equations (5) and (6). The weights $w_{i}$ take real values between zero and one to vary the priority given to different zones.

#### 2.2.2. Coverage Objective

The indoor positioning system should be able to provide the localization of a target in any part of the ROI. When estimating a 2D position with RDOA, it is necessary to acquire at least two distance-difference measurements that provide two intersecting hyperbolae. Thus, we need at least three distance measurements to perform localization. The target must therefore be within the scope of at least three sensors, and when this condition is satisfied, the target is considered to be three-covered. To ensure a high degree of coverage for localization, we need to optimize the *k*-coverage of the ROI, where k=3 in 2D localization and k=4 in a 3D scenario. We consider that a point of the ROI is covered by a sensor if the point has an LOS connection with the sensor, and we denote with $N_{k}$ the number of points of the grid that are *k*-covered. Maximizing the division of this number by the total amount of test points *P* increases the percentage of the ROI that is *k*-covered:

(9) $$f_{k-\text{cov}}=\frac{N_{k}}{P}$$

## 3. Proposed Algorithm

At the beginning of this section, we describe the problems that we have encountered when applying NSGA-II to the sensor placement problem. Then, we introduce and justify the modifications that we propose for NSGA-II. We have used the DEAP framework available for Python [40] to implement an evolutionary algorithm based on NSGA-II and to obtain the results that we present in Section 4.

NSGA-II starts ranking the population and assigning the crowding distance to the individuals. The algorithm compares the values that the individuals achieve after evaluating the objectives. An individual dominates another individual if all of the evaluated objectives of the former are better than those of the latter. Individuals that are not dominated by any other individual belong to the first front, which is called the PF. Individuals that are only dominated by those individuals of the PF are assigned to the second front, and so on. Once the population is ranked, the crowding distance is computed for each individual of the same front. The crowding distance is an estimation of the perimeter of the cuboid formed by an individual and its immediate neighbors of the front in the objective space. Among the individuals of the same front, NSGA-II prefers those with a higher crowding distance, so as to keep diversity within the population. After the assignation of non-dominance and crowding distance, the algorithm continues with the usual steps of a genetic algorithm (GA), namely, selection, crossover and mutation. When selecting the parents, the individuals compete in a tournament selection. The algorithm selects the individual of the lowest front. In case they belong to the same front, the individual whose crowding distance has a higher value is preferred. Crossover and mutation operators create a new offspring population, which is merged with the original population. Finally, this bigger population is reduced to the size of the original population after reassigning non-dominance and crowding distance. The reader can go deeper into NSGA-II through the aforementioned work [28].

**Figure 1:**
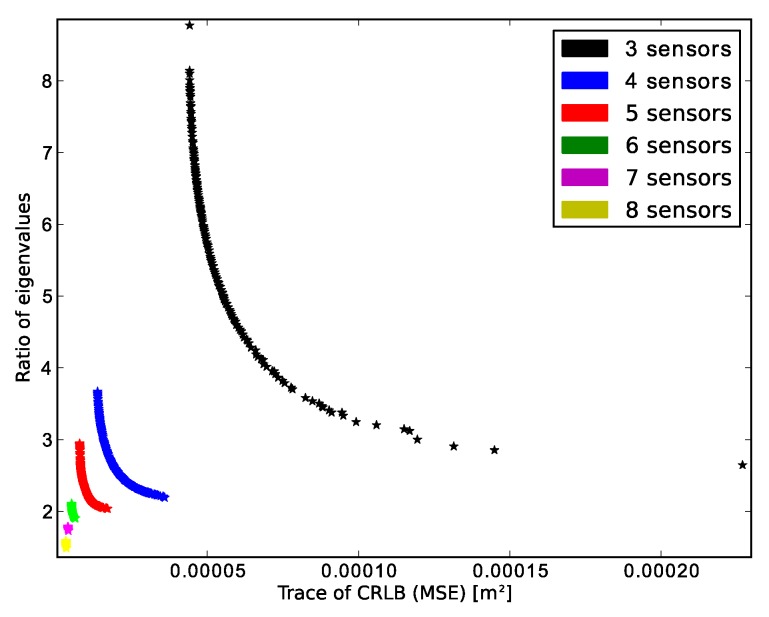
Pareto fronts of two uncertainty metrics for different numbers of anchor nodes.

In [29], we used the algorithm out-of-the-box as it is implemented in MATLAB for deploying a fixed number of anchor nodes. The obtained results were satisfactory; however, when applying the algorithm for deploying a variable number of anchor nodes, we do not obtain a full PF. We should be able to obtain the same solutions that we would get running the algorithm for different fixed numbers of anchor nodes independently. Of course, the algorithm must discard those solutions that achieve a lower accuracy even though their number of sensors is higher. After running NSGA-II for deploying three to eight sensors in a regular square, the obtained solutions are biased towards the lowest number a sensors. Figure 1 is useful for explaining this tendency.

The graph shows the PFs for different number of sensors after optimizing the MSE and the spread of the error ellipsoids. The solutions have been obtained with independent runs of NSGA-II varying the number of sensors. Since each PF has the same number of points, it can be seen that the crowding distance of solutions with three sensors will be higher. The tournament selection of NSGA-II uses the crowding distance as a mechanism to keep the variety of the individuals within the population. It is well known that for many-objective optimization, the solution’s convergence of NSGA-II is usually biased, and some researchers have proposed some mechanisms for diversity management [41,42]. Among those mechanisms, evolving multiple subpopulations independently allows one to keep diversity and improves the performance of the evolutionary algorithm [43]; this process is known as speciation or niching. After analyzing Figure 1, it is evident that there is a wide gap in the fitness landscape according to the number of sensors. Taking this issue into account, it seems to be a good idea to consider that those topologies with the same number of sensors belong to the same specie. Additionally, we use the coordinates of the anchors with real numbers as chromosomes; hence, crossover operations between individuals with different number of sensors become cumbersome, and that is another reason for speciation. We also have the benefit of being able to generate new topologies using structural mutation and migration, so that a topology formed after adding or removing a sensor competes with other topologies of the same number of sensors. We took this idea from [27], where the authors deploy a wireless sensor network optimizing coverage, connectivity and energy. However, they do not focus on accuracy.

**Figure 2:**
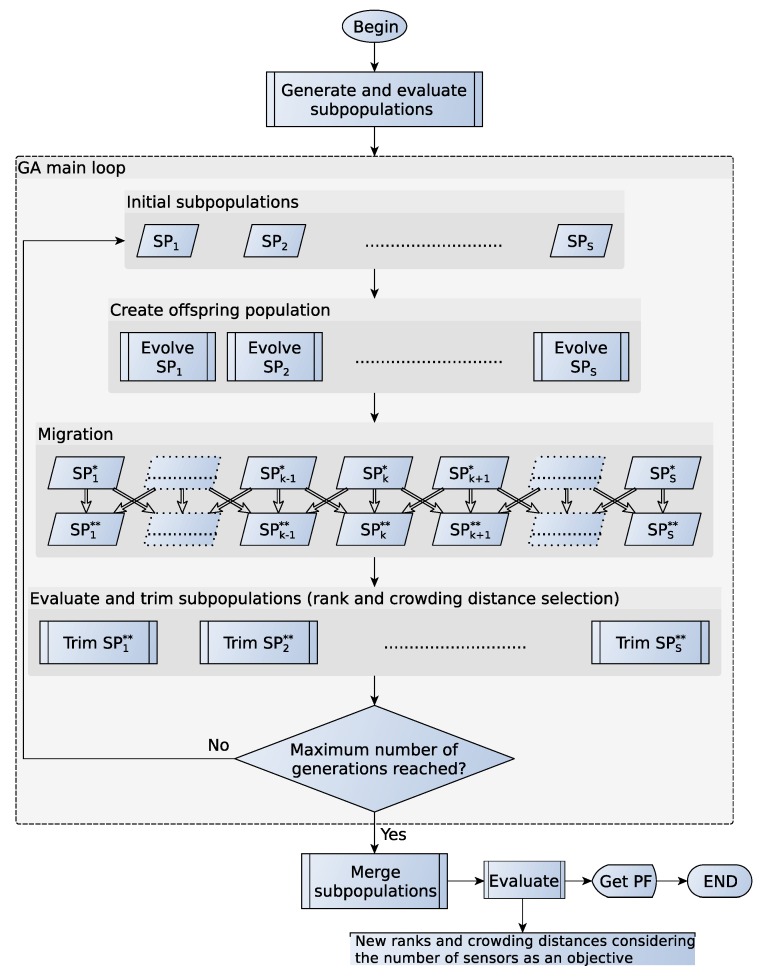
Proposed GA.

Figure 2 provides a general overview of the proposed GA. The algorithm starts creating *S* subpopulations ${\mathrm{SP}}_{k}$ with the same size $S_{p}$. The index *k* of the subpopulations takes values in the range [1,S]. The minimum and maximum number of sensors are constant and belong to ${\mathrm{SP}}_{1}$ and ${\mathrm{SP}}_{S}$, respectively. The individuals of ${\mathrm{SP}}_{2}$ have exactly one more sensor than ${\mathrm{SP}}_{1}$, and so on. The independent evolution of species also allows one to parallelize the creation and evolution processes. To summarize, the proposed GA is a basic NSGA-II where the population is split by the number of sensors. The subpopulations evolve concurrently, and it is possible that the amount of sensors varies due to the mutation operator. A structural mutation involves the addition or removal of a sensor. In case this happens to an individual of ${\mathrm{SP}}_{k}$, a migration process moves the individual to ${\mathrm{SP}}_{k+1}$ if addition or to ${\mathrm{SP}}_{k-1}$ when a sensor is removed. Each subpopulation undergoes four states during an iteration of the GA:

- ${\mathrm{SP}}_{k}$: initial population. Population size: $S_{p}$.
- ${\mathrm{SP}}_{k}^{*}$: evolved population, the initial population and its offspring. It can contain individuals with different number of sensors. Population size: $2S_{p}$.
- ${\mathrm{SP}}_{k}^{**}$: evolved population without individuals with different numbers of sensors. Population size varies among subpopulations.
- Trimmed ${\mathrm{SP}}_{k}^{**}$: best individuals of ${\mathrm{SP}}_{k}^{**}$ according to NSGA-II selection; i.e., non-dominance rank and crowding distance. It becomes the initial population in the next GA iteration. The size of the population is $S_{p}$.

We only evaluate the objectives, and assign ranks and crowding distances, of the individuals after creating the initial population and at the beginning of the trimming step. Once the algorithm has iterated over *G* generations, the resulting subpopulations are merged, and the algorithm returns the PF. We do not consider the number of sensors as an objective during the GA iterations. However, after merging all of the subpopulations, we perform another NSGA-II selection operation, including the number of sensors as a function to be minimized. This allows one to discard those solutions that achieve lower accuracy and coverage with more sensors; since they can be dominated by some individuals of a species with a lower index.

Finally, we describe the genetic operators that we use to create the offspring population. The remaining aspects of the GA are common or should have already been clarified. Since the beginning of the evolution step and just before the migration process, we apply a sequence of four genetic operators to the individuals of each subpopulation:

- Selection: tournament selection of two individuals. It selects $S_{p}$ individuals, which will be the parents of the offspring subpopulation. Two individuals are picked randomly among the initial subpopulation. The method checks first the non-dominance rank of each individual and selects the best one; in case they belong to the same front, it chooses the one with the higher crowding distance.
- Crossover: blend crossover. For each consecutive pair of two parents ($p_{i}$ and $p_{i+1}$) of the list given by the previous step, there is an $r_{x}$ chance of generating two new individuals ($c_{i}$ and $c_{i+1}$) that replace the parents; otherwise, they remain unchanged. Equation (10) gives the new individuals:
  (10) $$\begin{array}{cc} c_{i} & =\left(1-\alpha \right)\circ p_{i}+\alpha \circ p_{i+1}\\ c_{i+1} & =\alpha \circ p_{i}+\left(1-\alpha \right)\circ p_{i+1} \end{array}$$
  In our case, c and p are the coordinates of each sensor of the individual in a consecutive array. The variable *α* is a vector of random elements with the same length of p, and it takes random values in the range [-1,2]. The operator ∘ is the Hadamard (element-wise) product. The offspring can therefore be in the expanded cuboid formed by the parents. In case any sensor falls into any obstacle, the algorithm sends them to the nearer corner, so that they cover a bigger space without altering their position too much.
- Mutation: Gaussian mutation. There is an $r_{m}$ chance that an individual undergoes mutation. If mutated, a random variable $v∼(\mu_{m},\sigma_{m})$ is added to each gene of the individual; *i.e*., to a coordinate. We perform again the same procedure if the sensor falls into an obstacle.
- Structural mutation: at a given chance $r_{s}$, a sensor can be added to or removed from the individual. If the individual belongs to the first or last subpopulation, we can only add or delete a sensor, respectively; otherwise, the operation is randomly selected. When deleting, we compute the number of sensors that are LOS with each other and delete the sensor with the higher value. When there are two or more sensors with the same number of LOS sensors, we compute the sum of the distance from each one of these sensors to its LOS neighbors. The sensor with the lower value is then removed. The sensor that is nearer to the others should be the sensor that adds less information, since very close sensors result in almost overlapping hyperboloids. This deletion mutation allows one to obtain a sensor placement scheme with less sensors without sacrificing a good deal of accuracy and coverage. On the other hand, when adding a new sensor, we compute the k-coverage level for each grid point; as defined in Section 2.2. We place the sensor in the point of the grid with the lowest k-coverage level; in case two or more grid points share the same value, we choose the one with the higher sum of the distances from the grid point to its LOS sensors.

## 4. Results

**Figure 3:**
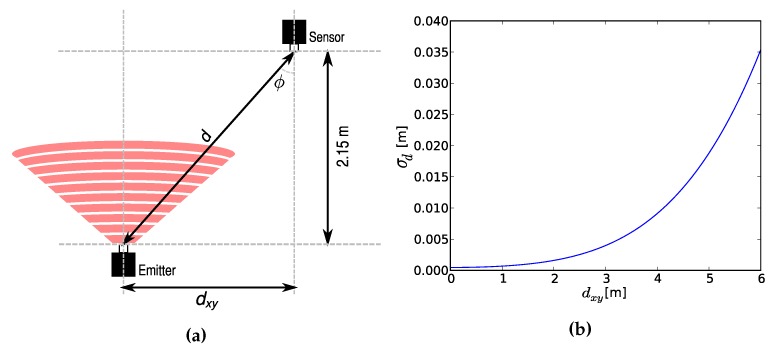
Infrared sensor-emitter geometry (**a**); and the standard deviation of the distance measurement as a function of the distance in the xy-plane (**b**).

We use the distance measurement model of an infrared system to get the variances of Equation (4). As shown in Figure 3a, the emitter moves along the xy-plane, while the height between emitter and sensor is constant. The distance error is a function of the distance between emitter and sensor $d=\sqrt{d_{xy}^{2}+2.15^{2}}$, as well as the angle of incidence $\phi =\mathrm{atan}\left(\frac{d_{xy}}{2.15}\right)$. We will not repeat the other constant parameters that model the system, which can be found in the works that describe it [44,45]. Finally, Figure 3b shows the evolution of the standard deviation of the distance measurement error $\sigma_{d}$ versus the distance in the xy-plane $d_{xy}$. Since we know the height of the emitter, we perform 2D localization in the horizontal plane.

We use the same probabilities for the genetic operators during the simulations that appear in the following subsections. Table 1 shows these values.

After each test, we provide the average execution time of each iteration of the algorithm. The code has been run on an Intel^®^ Core™ i7-4712MQ mobile processor with 8 GB RAM DDR3 1333 MHz (MSI CX61 2PC-1215XES, New Taipei City, Taiwan).

Putting it all together, we define a model of the ROI and a grid of points, which are the candidates for the location of the target. Our approach evaluates the objectives using these points as the position of the target, and then, it obtains a metric related to the whole area (e.g., the average accuracy). The algorithm starts generating a random population, sets of sensors placed in the ROI, and evaluating the objective functions for each individual. To compute the accuracy metrics, which are based on CRLB, we use the infrared model to obtain the covariance of the distance measurements and get the value of Equation (2). After evaluating the population, the algorithm starts iterating until a given number of generations is reached. Finally, it returns the PF.

### 4.1. LOS Sensors

**Figure 4:**
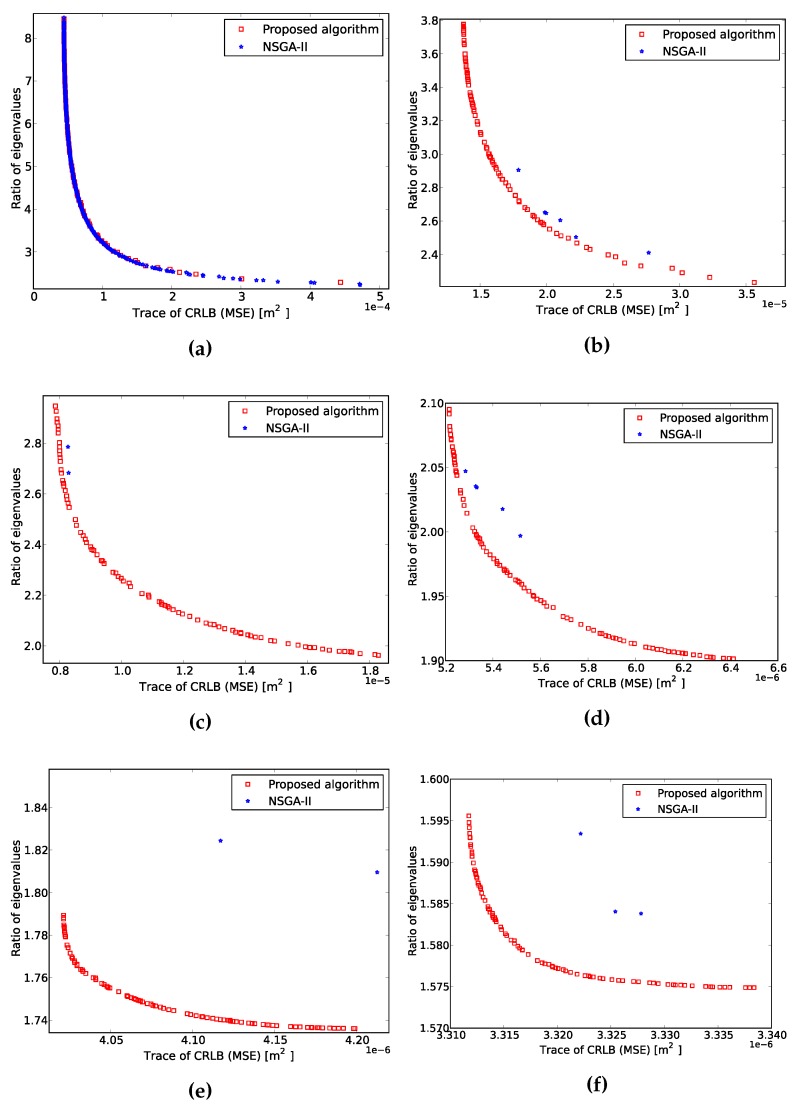
Comparison of Pareto fronts with non-dominated sorting genetic algorithm (NSGA-II) and the proposed algorithm. (**a**) Three sensors Pareto front; (**b**) four sensors Pareto front; (**c**) five sensors Pareto front; (**d**) six sensors Pareto front; (**e**) seven sensors Pareto front; (**f**) eight sensors Pareto front.

These results show the Pareto front for a sensor placement case when there is always an LOS path between the emitter and the sensors. It is the same case studied in [29] without fixing the number of sensors to a scalar constant. We consider six subpopulations, and the amount of sensors varies in the range of three to eight. Each subpopulation contains 100 individuals. The ROI is a $9\;m^{2}$ regular square, and we evaluate 121 positions regularly separated by 3cm and equally weighted. The Pareto front with NSGA-II has been obtained with a population of 600 individuals, where only individuals with the same number of sensors can perform crossover. We have run the algorithm for 2000 generations. The average time per iteration of the algorithm was 12 s. Figure 4 evidences the benefits and necessity of speciation.

The Pareto front has been split into six graphs according to the number of sensors. Each algorithm returns the same amount of Pareto solutions. As stated above, the solutions obtained with NSGA-II tend to the lowest amount of sensors, whereas the proposed algorithm can control the amount of solutions of each subpopulation. Hence, it converges to a Pareto front close to that of Figure 1. Table 2 concludes this case study by providing a comparison of both algorithms.

### 4.2. Occluded Sensors

This section shows the application of the algorithm for the same cases of [30]. In that paper, the standard NSGA-II did not converge to a smooth Pareto front. In addition, it can be seen that many solutions provide a high position error. The new algorithm provides a better convergence, as can be seen in the following results. We include a comparison with random deployment to show the benefits of using a deployment algorithm. The values of the objectives with random deployment have been obtained averaging 50 random sensor sets for each number of sensors. Instead of considering a coverage radius, we consider that a point is in the scope of a sensor if there is a straight line that joins them without crossing any obstacle. The degradation of the accuracy in points that are not near a sensor is taken into account in the measurement error model, as was shown in Figure 3b.

#### 4.2.1. Case 1: One Obstacle

**Figure 5:**
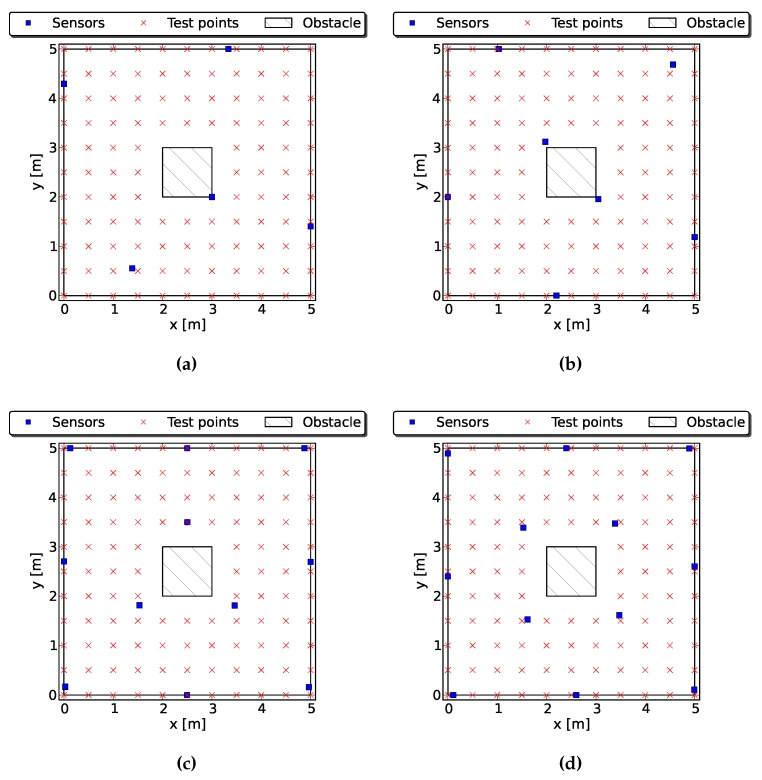
Some optimal configurations found by the proposed algorithm when deploying five to 12 sensors with an obstacle in the center. The whole ROI is at least three-covered in all cases. (**a**) Best solution with five sensors; (**b**) best solution with seven sensors; (**c**) best solution with 11 sensors; (**d**) best solution with 12 sensors.

This case study focuses on the placement of five to 12 sensors in a $25\;m^{2}$ regular square with an obstacle in the center. The obstacle is a $1\;m^{2}$ square column. We evaluate a total of 112 grid points that are regularly separated by 0.5m and equally weighted. The objectives to be optimized are the averaged trace of the CRLB and the percentage of the points that are at least three-covered. Each subpopulation contains 100 individuals, and the algorithm was run for 2000 generations. Each iteration of the algorithm took 55 s on average. Figure 5 shows some Pareto optimal solutions, and a comparison of our algorithm with random deployment can be seen in Table 3.

Our algorithm outperforms random deployment considerably, and the lower the number of sensors, the higher the evidence. The higher coverage is achieved with any amount of sensors. With a low number of sensors and the presence of occlusions, it may easily happen that some points of the ROI are only in the scope of collinear sensors. In case this happens, the accuracy of the target localization in these points will be very poor; see Table 3, five sensors with random deployment. As shown in Figure 5, the symmetry of the solutions is also a good indicator of the algorithm’s performance.

#### 4.2.2. Case 2: Two Obstacles

**Figure 6:**
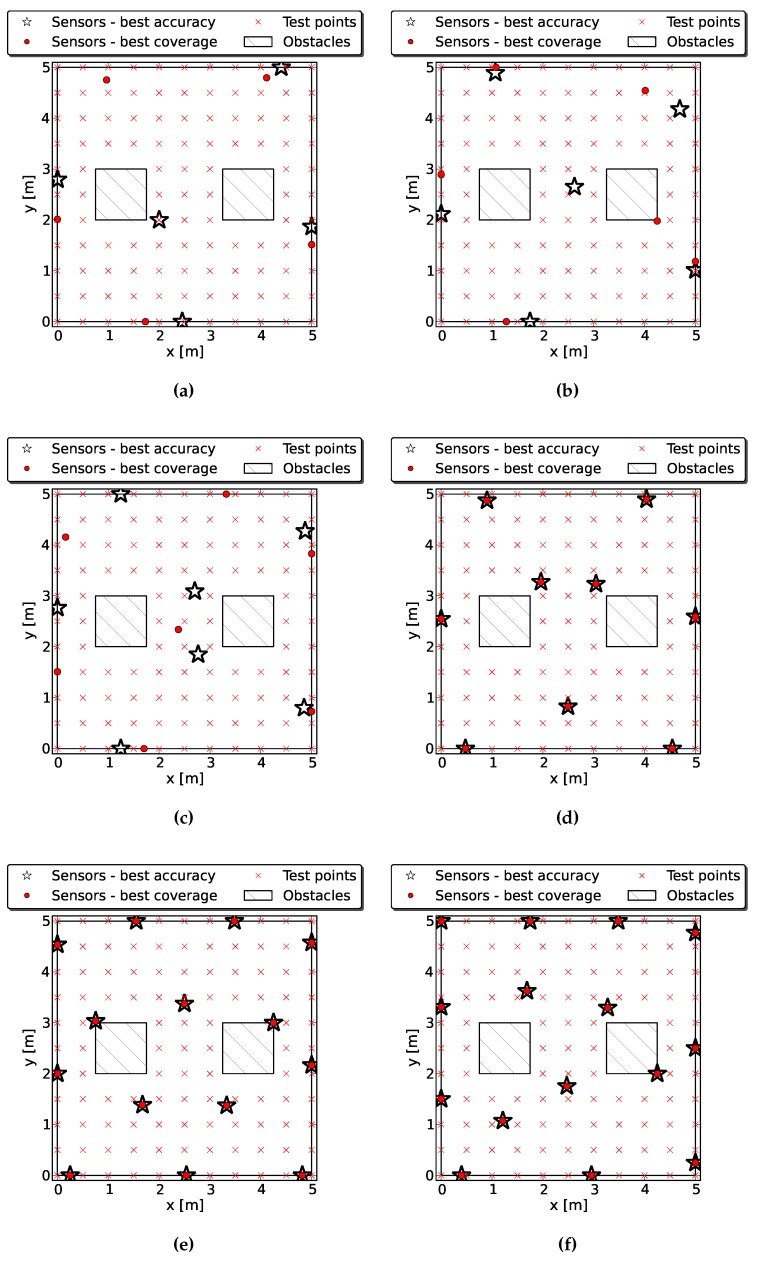
Some optimal configurations found by the proposed algorithm when deploying five to 15 sensors with two obstacles. The first three figures show optimum coverage and accuracy deployment, whereas the two objectives are simultaneously optimized in the other cases. (**a**) Best solutions with five sensors; (**b**) Best solutions with six sensors; (**c**) Best solutions with seven sensors; (**d**) Best solution with nine sensors; (**e**) Best solution with 14 sensors; (**f**) Best solution with 15 sensors.

In this example, we place five to 15 sensors in a room with the same shape considering the presence of two obstacles. The obstacles are again $1\;m^{2}$ square columns; their center points are [1.25, 2.5] and [3.75, 2.5]. The subpopulations can contain 20 individuals. The algorithm has been run for 1000 generations, and each iteration took 30 s on average. Figure 6 shows some Pareto optimal solutions. The full coverage is not always achieved with the best accuracy, and we show the extreme solutions of the Pareto front in these cases. When the solution with the best accuracy provides full coverage, the Pareto front is made of a single solution, and there is no actual trade-off. The configurations show a certain degree of symmetry; some basic shapes can also be recognized. Table 4 provides a comparison with random deployment. We only show the worst values of the Pareto front for each objective; the improvement is evident.

Finally, after analyzing these solutions, it can be seen that sensors are usually placed near the boundaries of the 5 m × 5 m square and the obstacles. We can consider an intuitive approach that places sensors in these boundaries regularly. However, this solution has probably the highest target-sensor distance; hence, it would be a good solution when the error due to distance between sensor and target is low or negligible. We must also take into account the fact that the area covered by a sensor decreases when the sensors are placed in the boundaries of obstacles. Nevertheless, we have evaluated this intuitive solution for the cases of one and two obstacles. We have placed sensors in the corners (internal and external squares, eight sensors for the first case and 12 sensors for the second). Placing eight sensors in the corners of the one obstacle case provides an average CRLB trace of 4.595e−5 $m^{2}$. Comparing this value to Table 3, it can be seen that we obtain a better accuracy with six sensors. When we place 12 sensors in the corners of the case with two obstacles, we get an accuracy of 1.533e−5 $m^{2}$, which can be improved with nine sensors according to Table 4. Anyway, this intuitive solution could also be a good starting point for generating the first population.

## 5. Conclusions

A multi-objective evolutionary algorithm for deploying a variable number of sensors for RDOA localization has been presented in this paper. We have acknowledged the strengths and weaknesses of state-of-the-art proposals on sensor placement for localization and proposed a method to deploy a variable number of sensors considering several objectives. Our results have shown a great improvement over random deployment in some NLOS scenarios. We have used speciation and structural mutations on NSGA-II, which is included in most standard libraries. The algorithm should therefore be easy to implement modifying these libraries. We have applied it on an infrared RDOA positioning system with a fairly complex measurement noise model. Additionally, using RDOA implies that the covariance matrix of the observations is not diagonal. Dealing with algebraic methods in such a system is quite hard or even unfeasible. However, with an evolutionary algorithm, we only need to evaluate the objectives without resorting to the computation of complicated derivatives. The structural mutations should improve the performance and speed of the algorithm. This can intuitively be seen with the shapes of some placement patterns. Deploying four sensors, we find the optimum configuration to be a square; whereas the configuration that optimizes the placement of five sensors is a square with a sensor in the center. It is evident that once we have obtained one of those solutions, we can obtain the other one with a structural mutation in a single step.

Future works will be oriented toward the reduction of errors due to multipath. As a result of reflections in walls, ceiling and floor, many reflected signals reach a sensor by different paths. This phenomenon is known as multipath interference. Multipath causes an offset in the distance measurement, which should be kept to a minimum. We are currently working on obtaining a model that determines the offset that reflected signals cause in the final measurement. The development and application of this model is an extremely difficult task since the multipath effect is highly dependable on the ROI. We should model the distance measurement as a sum of the true distance between sensor and target, the noise of the LOS signal and the contribution of the multipath (offset). The latter term will be another objective of the optimization problem. We will also focus on localization of targets in motion. Another line for future work is the improvement of the execution time of the algorithm exploiting the parallelization of modern multi-core processors and graphics processing units. As can be seen in Figure 2, the migration operator is the only part of the algorithm that cannot be parallelized, whereas evolution and trimming processes can happen concurrently. The different simulations shown in this paper took from three hours up to a full day running on a laptop. A comparison with other algorithms is also a subject of further studies.

## Figures and Tables

**Table 1:** Probabilities of the genetic operators.

Operator	Symbol	Probability
Crossover	$r_{x}$	0.80
Mutation	$r_{m}$	0.10
Structural mutation	$r_{s}$	0.05

**Table 2:** Extreme values of the Pareto fronts with NSGA-II and the proposed algorithm.

Algorithm	Amount of Sensors	Amount of Solutions	Lowest CRLB Trace (m^2^)	Lowest Ratio of Eigenvalues
**NSGA-II**	3	573	4.4085e−5	2.0723
	4	9	1.7882e−5	2.4111
	5	3	8.2704e−6	2.6833
	6	7	5.2845e−6	1.997
	7	4	4.117e−6	1.8098
	8	4	3.3222e−6	1.5838
Proposed algorithm	3	100	4.4083e−5	2.0397
	4	100	1.3753e−6	2.2331
	5	100	7.8631e−6	1.9624
	6	100	5.2156e−6	1.9014
	7	100	4.0213e−6	1.7361
	8	100	3.3117e−6	1.5749

**Table 3:** Comparison of the Pareto optimal solutions obtained by the proposed algorithm and random deployment for the case with one obstacle. The values for random deployment have been obtained averaging 50 randomly placed sets of sensors for each amount of sensors.

Amount of Sensors	Proposed Algorithm		Random Deployment	
	CRLB Trace (m^2^)	Coverage	CRLB Trace (m^2^)	Coverage
5	9.5475e−5	1	2.1418	0.9116
6	4.1925e−5	1	0.5002	0.9552
7	2.1706e−5	1	0.5471	0.992
8	1.2324e−5	1	0.0382	0.9875
9	9.4244e−6	1	2.3467e−4	0.9989
10	6.9933e−6	1	1.5574e−4	0.9989
11	5.7051e−6	1	1.1498e−4	0.9996
12	4.7841e−6	1	6.4065e−4	0.9998

**Table 4:** Comparison of the worst Pareto values obtained by the proposed algorithm and random deployment for the case with two obstacles. The values for random deployment have been obtained averaging 50 random placed sets of sensors for each amount of sensors.

Amount of Sensors	Worst Pareto Values with Proposed Algorithm		Random Deployment	
	CRLB Trace (m^2^)	Coverage	CRLB Trace (m^2^)	Coverage
5	2.0486e−4	0.7523	0.2175	0.7593
6	9.2725e−5	0.9817	0.5072	0.8930
7	3.2163e−5	0.9817	0.0178	0.9349
8	1.5879e−5	1	0.0123	0.9624
9	1.2524e−5	1	0.009	0.9679
10	9.4426e−6	1	2.9721	0.9811
11	7.0657e−6	1	0.3861	0.9868
12	5.8686e−6	1	8.2985e−4	0.9910
13	5.1978e−6	1	6.883e−3	0.9901
14	4.4578e−6	1	0.4218	0.9936
15	4.0501e−6	1	1.508e−4	0.9963
